# What does the media say about palliative care? A descriptive study of news coverage in written media in Spain

**DOI:** 10.1371/journal.pone.0184806

**Published:** 2017-10-02

**Authors:** José Miguel Carrasco, Miriam García, Alejandro Navas, Inés Olza, Beatriz Gómez-Baceiredo, Francesc Pujol, Eduardo Garralda, Carlos Centeno

**Affiliations:** 1 ATLANTES Research Program, Institute for Culture and Society, University of Navarra, Pamplona, Spain; 2 Navarra Institute for Health Research (IdiSNA), Pamplona, Spain; 3 Errea Comunicación, Pamplona, Spain; 4 Public Communication Department, School of Communication, University of Navarra, Pamplona, Spain; 5 GRADUN, Institute for Culture and Society, University of Navarra, Pamplona, Spain; 6 Journalism Projects Department, School of Communication, University of Navarra, Pamplona, Spain; 7 Department of Economics, School of Economics and Business Administration, University of Navarra, Pamplona, Spain; Rudjer Boskovic Institute, CROATIA

## Abstract

**Introduction:**

The goal of palliative care (PC) is to improve the quality of life of terminal stage patients and their families. The subject frequently appears in the *mass-media* and this helps create a socially accepted identity. The aim of this study is to describe and analyse PC related news items appeared in the Spanish written media.

**Methodology:**

A descriptive cross-sectional study was designed. Considering diffusion, scope and the range in editorial policy criteria, four printed newspapers (PN) were selected, together with four exclusively digital media sources (DM). Through *Mynews*, a newspaper content depository, and the search tool for each DM website, articles published between 2009 and 2014 which included the terms "palliative care" and "palliative medicine" were sought. A questionnaire was created to characterise each article identified and a descriptive analysis was undertaken.

**Results:**

A total of 627 articles were identified, of which 359 (57%) were published in PN (42% in the printed editions -PE- 16% in their online editions -OE-) and 268 (43%) in DM. In general, they appeared mainly in sections concerning *Health* (23%), *Culture and Society* (18%) and *General/Home News* (15%). In PE, just 2% were found in the *Health* section and nearly 70% in *Culture and Society* and *General/Home News*. Most of the articles were informative in nature and contained socio-political messages (90%). Statements by PC professionals were found in 35% of the articles and by politicians in 32%. The most frequent content was related to facing end of life (74%) and patient quality of life (70%).

**Conclusions:**

The Spanish written media reflects the socio-political interest aroused by PC. Nevertheless, messages circulating about PC do not describe professional practice, or the contribution of the same for patients. Content more in line with the clinical practice might help contribute to the development of this new area of medicine.

## Introduction

An aging population and the increased risk of developing neurodegenerative diseases and cancer are the consequences of demographic and epidemiological changes which have taken place in recent decades, especially in developed countries [1,2]. It is forecast that the coming years will see an increase in the prevalence of chronic and advanced-stage diseases, which will also lead to longer periods of illness [3]. From a Public Health approach managing the social and healthcare implications of those changes poses a major challenge. These are happening so fast that the generation which identifies them will also suffer their consequences.

Palliative Care (PC) has been defined by the World Health Organisation (WHO) as "*an approach that improves the quality of life of patients and their families facing the problem associated with life-threatening illness*, *through the prevention and relief of suffering by means of early identification and impeccable assessment and treatment of pain and other problems*, *physical*, *psychosocial and spiritual"* [4]. International organisations, such as the European Association for Palliative Care (EAPC) and the WHO, have repeatedly stated the importance of developing and integrating PC into healthcare systems [2,5], and the deficient coverage at this time [6]. In Spain, for the year 2011 it is estimated that there were 500 deaths per 100,000 inhabitants due to illnesses which had terminal stages [7] and that PC coverage in 2012 was 50% lower than the recommended level [6]. The discipline's youth and its insufficient development limit the general population's knowledge of the same. Furthermore, sociocultural taboos related to death in the Spanish context may condition the social perception of end of life care [8,9].

Several studies have identified the lack of knowledge and awareness about PC as key obstacles to its development [10,11]. Some evidence may be found in the literature identifying the newspapers as one of the main sources of information on PC, and the mass-media as key in delivering messages about them [12]. The mass-media have the aim of informing, and are a key agent for socialisation and in generating public opinion; they represent the main means of transfer of ideas in the public space [13] and its influence should not be underestimated when constructing the social image of PC [14,15].

The role of communication in the field of health has been studied in some depth, especially those factors related to health promotion and disease prevention [16–18]. Some studies have examined the relationship between the media and specific health problems or healthcare policy [19–22] but notwithstanding this, the presence and impact of PC in the media has hardly been studied. In PC, communication has been studied in its internal aspect, which is to say, communication between multidisciplinary team PC healthcare professionals and between these experts and the patient and their family members [23,24]. The scientific literature shows how promotional campaigns in the mass-media may help to promote PC both from a social and from a healthcare point of view [25].

A critical examination of written media may generate knowledge and evidence contributing to understand how palliative care is perceived by the society, and helping to develop strategies for its promotion and development. Based on these considerations, this study aims to describe and to critically analyse PC related news items which have appeared in recent years in the Spanish written media.

## Material and methods

In order to reach the proposed aim, a descriptive cross-sectional study was designed.

Considering diffusion, scope and range in editorial policy criteria, four printed newspapers (PN: *El País*, *El Mundo*, *ABC*, *La Vanguardia*) were selected, together with four exclusively digital media sources (DM: *Lainformacion.com; Publico.es; Libertaddigital.com*, *Elconfidencial.com*). All of them are national newspapers directed to Spanish audience. Using Mynews (www.mynews.es), a domestic newspaper content repository, and the search tool for each DM website, articles published between January 2009 and February 2014, in any format (printed, online, regional or national) which included the terms “cuidados paliativos” and/or “medicina paliativa” (in English "palliative care" and/or "palliative medicine") were sought. As regards newspapers, when the printed edition (PE) and the online edition (OE) contained the same news item, PE was chosen.

Due to the lack of a developed and validated questionnaire to characterise news related to PC, a purpose-built questionnaire was developed by a team of eight professional experts in journalism, communication, linguistics, sociology, economics, public health and PC. The questionnaire was developed with the aim of characterising each news item found, compiling information on: the type of story, the tone, type of language used, the sources used, the target group for the item, its position within the page and the nature of the content and specific issues on PC and end of life care (S1 File). A first draft of the questionnaire was developed by the first and the second author, and it was sent by email to the other authors to add comments and suggestions based on their own knowledge in order to define each questionnaire's item and its categories. With the feedback received, a preliminary version was developed, and discussed and agreed in a face to face meeting. This version was tested in a random sample of 50 news and after minor changes, the definitive version was sent to all the team members to reach final approval.

Two journalists undertook the search and identified the news items. Whenever possible, the format used was Portable Document Format (pdf). The journalists applied the questionnaire to each identified news item and the results were registered on an online form, designed using Surveymonkey (https://es.surveymonkey.com). A database was created by the first author to include the information extracted from the forms, checking the codification process and refining inconsistencies. In case of disagreement in the codification given to an item, it was discussed with the journalists until a consensus was reached. The statistical analyses were performed with the Stata 12 software package [26]. Differences between proportions were tested using the Chi Square test and the Fisher exact test, taking as statistically significant those values of p<0.05.

## Results

A total of 627 articles were identified over the five years of the study. Just over half (359 articles, 57%) appeared in newspapers (42% in the PE, 16% only in the OE). A further 268 articles (43%) were identified in exclusively digital media (Table 1).

**Table 1:** Origin of identified articles.

	n
**Printed newspaper**	**359 (57.3%)**
*Printed editions*	*262 (41*.*8%)*
ABC	135
El País	65
El Mundo	24
La Vanguardia	38
*Online editions*	*97 (15*.*5%)*
El Mundo	57
La Vanguardia	40
**Digital media**	**268 (42.7%)**
Elconfidencial.com	6
Lainformacion.es	203
Libertaddigital.com	18
Publico.es	41
**Total**	**627**

The greatest number of PC related news items were identified in sections concerning *Health* (23%), followed by *Culture and Society* and *General/Home News* (18% and 15% respectively), with minor appearances in *Editorial and letters* (9%). When looking exclusively at PE newspaper editions, significant variations were observed: 38% were seen in *Culture and Society*, *30% in General/Home News*, almost a fifth in *Editorial and Readers’ letters* (19%) and just 2% in *Health* (Table 2).

**Table 2:** Section where articles appear.

	Total		*Printed edition*	
	N = 627*		N = 262*	
	n	%	*n*	*%*
Health	142	22.6	*4*	*1*.*5*
Culture and/or Society	114	18.2	*100*	*38*.*2*
General / Home News	95	15.2	*78*	*29*.*8*
Editorial and letters	58	9.3	*50*	*19*.*1*
Obituaries	6	1.0	*5*	*1*.*9*
Other / Unidentified	212	33.8	*25*	*9*.*5*

* Significant statistical differences are observed between the different media.

The majority of the documents identified were informative and objective, that is to say, they stated facts and ideas to bring them into the public domain (86%). A quarter of them, half in the case of PE (52%), included value judgements and subjective interpretations. The type of message transmitted was predominantly socio-political (90%), with the capacity to generate opinion and debate, with a smaller professional, healthcare component and practically no scientific content (5%). The main target group for the documents identified was the general public (95%). The tone and language used in the documents was typically neutral (86%) and easy to understand (95%). The main journalistic hooks to attract the attention of readers were human interest in PC (77%) and associated socio-political factors (72%). Other hooks were the concept of "progress" (40%) and allusions to controversial issues (39%), with significant differences between the diverse editions. Statistically significant differences were found between media sources, except in the case of the socio-political message (Table 3).

**Table 3:** Features of identified articles.

	TOTAL		Printed newspaper				Digital media		
	N = 627		Printed editions N = 262		Online Editions N = 97		N = 268		
	n	%^a^	n	%^a^	n	%^a^	n	%^a^	p
**TYPE OF DOCUMENT**^b^^,^^c^									
Informative	541	86.3	212	80.9	92	94.8	237	88.4	*
Interpretative	153	24.4	136	51.9	5	5.6	12	4.5	*
Educational	246	39.2	3	1.1	77	79.4	166	61.9	*
Complaint	207	33.0	94	35.9	15	15.5	98	36.6	*
Enquiry	231	36.8	-	-	34	35.1	197	73.5	*
**TYPE OF MESSAGE**^b^^,^^d^									
Scientific	31	4.9	20	7.6	6	6.2	5	1.9	*
Healthcare	412	65.7	142	54.2	77	79.4	193	72.00	*
Professional	386	61.6	123	46.9	74	76.3	188	70.1	*
Socio-political	566	90.3	250	95.4	93	95.9	223	83.2
Other	75	12.0	3	1.1	72	74.2	-	-	*
**TARGET GROUP**									*
Family members	3	0.5	-	-	3	3.1	-	-
PC Professionals	14	2.2	3	1.1	1	1.0	10	3.7
Healthcare professionals	3	0.5	1	0.4	-	-	2	0.7
General public	598	95.4	252	96.2	92	94.8	237	88.4
**TONE**									*
Emotional	67	10.7	52	19.8	8	8.2	7	2.6
Neutral	538	85.8	207	79.0	88	90.7	243	90.7
**LANGUAGE**									
Educated	4	0.6	4	1.5	-	-	-	—
Common	598	95.4	252	96.2	96	99.0	250	93.3
**JOURNALISTIC DOCUMENTATION**									*
Appropriate	559	89.2	255	97.3	77	79.4	227	84.7
**JOURNALISTIC HOOKS**^b^									
Human interest	482	76.9	200	76.3	74	76.3	208	77.6
Socio-political	449	71.6	151	57.6	67	69.1	231	86.2	*
Progress	249	39.7	7	2.7	55	56.7	187	69.8	*
Conflict	246	39.2	91	34.7	28	28.9	127	47.4	*
Unusual	109	17.4	-	-	47	48.5	62	23.1	*
Fame	53	8.5	32	12.2	13	13.4	8	3.0	*
Other	57	9.1	3	1.1	50	51.5	4	1.5	*

^**a**^ % calculated of N total.

^b^ Non-exclusive categories.

^c^ Type of document. Informative: states facts and ideas to present them to the general public; Interpretative: offers judgements and subjective interpretations; Educational: seeks to teach in a clear, concise manner; Complaint: represents a protest or social problem; Enquiry: provides guidance or reference for scientific or technical issues.

^d^ Type of message transmitted. Scientific: informs of data, findings or scientific evidence in the field of PC; Care: refers to the provision of medical, pharmaceutical, psychological or other services. Professional: reference to the aspects and implications of PC as a specific professional sector; Socio-political: reference to socio-political aspects or debates.

* Significant statistical differences are observed between printed and digital media.

Statements by doctors were frequently featured in documents (35%), but these vary in proportion: in PE they can be found in 10% of the total, whilst in online editions and digital media, they appear in over half the total. Statements from politicians was the second most frequently seen (32%) and can be identified in a quarter of the printed documents and in greater proportion in online editions and digital media (43% and 36% respectively). Accounts from patients, relatives and scientists hardly exist. The use of data and figures to qualify information was more frequent in digital media (84%) than in printed formats (Table 4).

**Table 4:** Origin of statements, data and bibliographical references presented in articles.

	TOTAL		Printed Media				Digital media	
	N = 627		Printed editionsN = 262		Online editions N = 97		N = 268	
	n	%^a^	n	%^a^	n	%^a^	n	%^a^
Doctors	221	35.2	26	9.9	52	53.6	143	53.4
Politicians	201	32.1	63	24.0	42	43.3	96	35.8
Patients	25	4.0	12	4.6	5	5.2	8	3.0
Family members	22	3.5	11	4.2	8	8.2	3	1.1
Scientists	16	2.6	10	3.8	-	-	6	2.2
Data and figures	333	53.1	54	20.6	54	55.7	225	84.0
Bibliographic references	24	3.8	10	3.8	3	3.1	11	4.1

^**a**^ % calculated of N total.

Content related to patient quality of life (70%), facing end of life (74%) and the importance of integral PC attention (68%) were the most frequently identified in the articles. Differences can be seen between different formats. In over half of the articles (64%), mention of PC was related to party political actions or manifestos. In 61% of the documents PC were linked to death, more commonly in digital media (88%) than in the online or printed editions (74% and 29%, respectively). A certain link was identified between references to PC and discussing issues related to euthanasia, with mentions in almost a quarter of printed newspapers and to a much lower degree in their online editions (7%) and digital media (3%). The links between articles which defend euthanasia were fewer in number than those which oppose it. The concept of "dignified death" was also frequently linked to PC (26%). Almost a quarter of the news items identified suggest the need to legally regulate PC (23%) and just under half suggest in some way, that there is a need to train and accredit professionals who provide PC (42%). Hardly any mention is made of the relationship between PC and opioids and of the need to rely on ethical committees in PC. Statistically significant differences were seen between all the newspapers. (Table 5).

**Table 5:** Topic contents identified in articles.

	TOTAL		Printed Media				Digital media		
	N = 627		Printed Editions N = 262		Online Editions N = 97		N = 268		p
	n	%^a^	n	%^a^	n	%^a^	n	%^a^	
**GENERAL**^b^									
Patient quality of life	441	70.3	126	48.1	70	72.2	245	91.4	*
Symptom and pain management	387	61.7	111	42.4	44	45.4	232	86.6	*
End of life	464	74.0	154	58.8	67	69.1	243	90.7	*
Terminal illness	392	62.5	101	38.5	59	60.8	232	86.6	*
Extension of PC to chronic illnesses	189	30.1	56	21.4	41	42.3	92	34.3	*
Family environment	166	26.5	70	26.7	31	32.0	65	24.3	*
**HEALTHCARE PRACTICE**^b^									
The importance of integral PC assistance	425	67.8	124	47.3	72	74.2	229	85.4	*
The importance of Professional-patient communication	301	48.0	26	9.9	61	62.9	214	79.9	*
The importance of giving greater importance to Primary Healthcare	234	37.3	10	3.8	49	50.5	175	65.3	*
The importance of giving greater relevance to Home Care	102	16.3	43	16.4	16	16.5	43	16.0
Helping the family during bereavement	108	17.2	20	7.6	28	28.9	60	22.4	*
**ANALGESIC OPIOIDS**^b^									
Addiction and side effects	5	0.8	1	0.4	1	1.0	3	1.1
Linked to death	49	7.8	13	5.0	3	3.1	33	12.3	*
Linked to suffering	50	8.0	32	12.2	3	3.1	15	5.6	*
**RELATIONSHIP BETWEEN DEATH AND EUTHANASIA**^b^									
PC is linked to death	384	61.2	77	29.4	72	74.2	235	87.7	*
PC is linked to euthanasia	77	12.3	61	23.3	7	7.2	9	3.4	*
PC is linked to the suspension of treatment	71	11.3	40	15.3	7	7.2	24	9.0	*
Euthanasia is being defended	36	5.7	21	8.0	7	7.2	8	3.0	*
Euthanasia is rejected	99	15.8	60	22.9	6	6.2	33	12.3	*
PC is linked to the concept of "dignified death"	165	26.3	93	35.5	12	12.4	60	22.4	*
**SOCIOECONÓMICAL ASPECTS**^b^									
PC as issues in party political programmes	403	64.3	132	50.4	85	87.6	186	69.4	*
Economic crisis and healthcare resources	63	10.0	6	2.3	5	5.2	52	19.4	*
**LEGAL ASPECTS**^b^									
The need for a law on PC	143	22.8	59	22.5	14	14.4	70	26.1
Writing advance directives	54	8.6	40	15.3	5	5.2	9	3.4	*
**PROFESSIONAL ASPECTS**^b^									
The need for training and professional accreditation in PC	265	42.3	27	10.3	35	36.1	203	75.7	*
Differences and/or lack of interprofessional agreement on PC	142	22.6	3	1.1	20	20.6	119	44.4	*
**ETHICAL ASPECTS**									
The need for ethical committees in PC	13	2.1	7	2.7	5	5.2	1	0.4	*

^**a**^ % calculated of N total.

^b^ Non-exclusive categories.

* Significant statistical differences are observed between printed and digital media.

## Discussion

This study collates and describes mentions made of PC in eight written media in Spain, between 2009 and 2014. The more than 600 documents identified make it very clear that there is an ongoing PC presence in the written media, which implies its relevance as part of ‘agenda setting’ [27]. Nevertheless, we have found that messages being circulated frequently do not accurately reflect the clinical practice and its nature.

The general public is the main target group for news items related to PC, predominantly informative and social in nature, including expert interventions in published articles. Social interest concerning PC is evident, with the presence of statements by politicians, links between the documents identified and party political proposals and the mention of PC in general news articles and readers' letters. These features may suggest the political use of PC to implicitly approach other issues, such as those related to euthanasia. Moreover, the fact that in our setting, death-related ideas have not been widely debated, nor socially assimilated [8,9] might well be a conditioning factor as regards positions taken when transmitting messages related to PC [28].

A 2011 study on the press showed the increase in access to the online press media as a current information source [29]. Another conclusion is that citizens look to newspapers in search of material to read on in-depth topics and analysis, which means that the newspaper have the power to generate trends and public opinion [29]. In respect to this, our data shows that how references to PC in the *Health* section in printed editions are practically testimonial and much less habitual than in sections devoted to *Culture and Society* and *General/Home News*. This means that PC is closer in nature to socio-political debates than to those discussing healthcare.

Formal factors (legal, accreditation, etc.) and those relating to structure (number of services and professionals, etc.) have both been identified as the main obstacles to the development of PC, but also to the lack of knowledge and of its social standing [10, 11, 30]. In Spain, as in other countries, existing knowledge about PC is scarce and insufficient [12].

The mass media plays a fundamental role in helping create a collective conscience as regards certain healthcare practice. On occasions the images they transmit may not coincide with reality and may generate unreal impressions about healthcare processes [31]. In spite of the scarcity of evidence available on the effect which information has on PC for people, some studies do offer information in this respect. A positive perception of PC translates into feelings of security which facilitate more appropriate end of life care; negative or erroneous perceptions create barriers to the professional-patient relationship [32].

A study carried out in Northern Ireland among the general population identified newspapers as the main source for providing information on PC for its citizens. Participants reported that the mass media was the main channel for spreading information on the same [12]. Other studies presented evidence on how the communication media might act as a vehicle to successfully disseminate information on strategies designed at overcoming the lack of knowledge and awareness of PC [25, 33].

In our study differences have been found in the number of articles in each of the media sources reviewed and statistically significant differences were present in practically all the comparisons carried out. This suggests a number of different approaches and areas to be focussed on regarding PC. Knowledge of the current diversity of news media, the features of the news they publish and of their readers, might help determine the most appropriate sources to disseminate news and adapt messages related to PC [15]. Participation of PC experts in related news and debates might help create a collective awareness, based on reality, rejecting sensationalism and misuse of the discipline [34].

In order to interpret the results correctly some methodological issues should be taken into account. Regarding the identification of articles, there is the risk that some were not detected since they may have discussed palliative care but without actually mentioning the terms "palliative care" or "palliative medicine". Furthermore, the exhaustiveness and sensitivity of the websites search tools cannot be guaranteed, and there is the risk that some articles could not be identified. Likewise, the lack of a validated questionnaire to characterise PC-related news may limit the descriptive capacity of the questionnaire used. In contrast, the large multidisciplinary team and the dynamics followed to define the items and the categories, are one of the strengths of the questionnaire, and could be a first step to validate a questionnaire. Having two experts carrying out the search and codification of the articles does not necessarily guarantee that these will be free from subjectivity, although it will help to minimise the impact. Furthermore, the results are sufficiently wide-ranging to minimise possible observer bias. Finally, eight of the most widely read printed news sources in our setting were chosen whilst those with lower circulation were not examined.

In order to contextualise the study period, during 2010 and 2011 three regional laws on end-of-life-care were discussed and passed [35, 36, 37], and no other was approved until 2015. It means that the studied period includes the first wave of attention on end-of-life care in the Spanish context.

To our knowledge, this project is the first to approach the study of the presence of PC in the news media. Our results help to understand the relationship of the mass media with regard to creating public opinion with respect to PC and the implications which this might suppose for its development. Understanding the public view of PC is necessary to design promotional strategies aimed at the appropriate management of the needs, expectations and resources regarding end of life care [33]. Although this study is focussed on the Spanish situation in a specific period of time, these results provide relevant information which might be applicable to other settings regardless of the time and the geographical context.

Further studies are needed to go deeper into the impact which newspaper texts on PC have on creating collective consciousness related to end of life care. An analysis of the format and content of these texts, based on qualitative and linguistic research, might provide more information on the subject. Moreover, whilst it is true that printed media influences the "agenda setting" of other communication media, the impact on generating public opinion from the TV and radio should not be overlooked as regards content featuring PC. The multidisciplinary team responsible for this study continues to pursue both lines of research.

## Conclusion

The Spanish written media reflects the interest aroused by PC but related news seems to be conditioned partly by socio-political debates related to how to address end of life care. A presence of PC in the news media, more in line with its definition, its clinical practice and with the contribution it might make to patient quality of life and of those around them, may serve to further its development and implementation.

## Supporting information

S1 File PC&Medias-S1-PlosOneV1.doc. Questionnaire items used in the study.
(DOCX)

S2 File PC&Mecias-S2-PlosOne-Reflist.docx. List of the articles included in the analysis.
(DOCX)
