# Supplementary material for: What does the media say about palliative care? A descriptive study of news coverage in written media in Spain
Source: PLoS One. 2017 Oct 2;12(10):e0184806. doi: 10.1371/journal.pone.0184806 (PMC5624582; doi:10.1371/journal.pone.0184806)
Supplement: S1 File — Questionnaire items used in the study. (DOCX) [file pone.0184806.s001.docx]

**S1 Questionnaire items used in the study.**

**General type of document** [Multiresponse]

**YES - NO** Informative-expositional. The aim is to inform and state facts and ideas.

**YES - NO** Interpretative or opinion. Offers judgment or subjective interpretations.

**YES - NO** Educational. Seeks to teach in a clear, concise manner.

**YES - NO** A complaint or exposure. With intent to demand change or social protest.

**YES - NO** Enquiry. Functions are a reference or guide to consult scientific or technical issues.

**Type of message transmitted.** [Multiresponse]

**YES - NO** Scientific

**YES - NO** Healthcare and/or management

**yes - NO** Professional.

**YES - NO** Social.

**YES - NO** Other.

**Main target group for the document**

**1** General Public [excluding direct messages to family members and/or patients].

**2** Professionals from the specific PC community: doctors, nurses, carers, psychologists, social workers, volunteers.

**3** Healthcare professionals in general.

**4** Patients.

**5** Family members.

**6** Others.

**Section where the document is found**

**1** Home News.

**2** Health.

**3** Culture and/or Society.

**4** Other

**Issues discussed in the article/video/audio** [Multirresponse]

**General concepts**

**YES - NO** Patient quality of life.

**YES - NO** Symptom or pain management.

**YES - NO** End of life.

**YES - NO** Terminal illness.

**YES - NO** Extension of palliative care to chronic illnesses.

**YES - NO** The patient's family environment.

**Healthcare practice**

**YES - NO** The importance of integral PC assistance .

**YES - NO** The importance of communication with healthcare workers/patients/family members.

**YES - NO** The importance of giving a greater or emphasis to primary healthcare.

**YES - NO** Patient care provided at the family home.

**YES - NO** Helping the family during bereavement.

**Opioids**

**YES - NO** Addiction and side effects due to opioid consumption.

**YES - NO** Opioid use is linked to death.

**YES - NO** Assimilation of the use of morphine and other opioids in the final stage.

**Death and Euthanasia**

**YES - NO** The association between PC and the idea of death.

**YES - NO** The association between PC and the idea of euthanasia.

**YES - NO** PC is linked to the suspension of treatment.

**YES - NO** Defence of euthanasia.

**YES - NO** Rejection of euthanasia.

**YES - NO** Concept of "dignified death".

**Socioeconomic aspects**

**YES - NO** PC as an issue in the programmes of political parties, the Government (Ministry of Health).

**YES - NO** Economic crisis and incidence in healthcare resources dedicated to PC.

**Legal aspects**

**YES - NO** The need for a law on PC.

**YES - NO** Writing up a will.

**Professional aspects**

**YES - NO** The need for training and professional accreditation in CP.

**YES - NO** Differences and/or lack of interprofessional agreement on PC.

**Ethical aspects**

**YES - NO** The need for ethical committees in PC.

**Statements and Resources** [Multirresponse]

**YES - NO** Contains statements from doctors.

**YES - NO** Contains statements from patients.

**YES - NO** Contains statements from family members.

**YES - NO** Contains statements from scientists.

**YES - NO** Contains statements from politicians.

**YES - NO** Contains bibliographical references.

**YES - NO** Contains data /figures.

**Tone**

**1** Neutral/Aseptic.

**2** Emotional**.**

**Type of Language**

1 Common, easy for everybody to understand.

2 Educated, includes some technical terms, aimed at those with a medium-high educational level.

3 Technical, with terminology, technicalities and acronyms which would only be understood by other professionals.

**Documentation from the journalistic point of view**

1. Seems to be appropiately documented.

2. Does not seem to be appropiately documented and distorts the reality of palliative care.

**The journalistic point of entry of the article/audio/video to attract readers' attention is... (Multiresponse)**

**YES - NO** Human interest.

**YES - NO** The important point includes political, social and economic factors.

**YES - NO** Progress.

**YES - NO** Conflict.

**YES - NO** Out of the ordinary.

**YES - NO** Famous people mentioned.

**YES - NO** Other.
