# Supplementary material for: What does the media say about palliative care? A descriptive study of news coverage in written media in Spain
Source: PLoS One. 2017 Oct 2;12(10):e0184806. doi: 10.1371/journal.pone.0184806 (PMC5624582; doi:10.1371/journal.pone.0184806)
Supplement: S2 File — List of the articles included in the analysis. (DOCX) [file pone.0184806.s002.docx]

**S2 List of the articles included in the analysis.**

**PRINTED NEWSPAPER**

***ABC***

- "En España aún mueren pacientes con unos sufrimientos evitables". (ABC; 17/01/2014)
- El Hospital Carlos III acogerá a los enfermos crónicos de media estancia. (ABC; 26/11/2013)
- Sí a la vida. (ABC; 04/04/2013)
- San Juan de Dios, una orden dedicada a los madrileños. (ABC; 16/03/2013)
- Los médicos envían a Chávez a casa por el avance de un nuevo tumor. (ABC; 01/03/2013)
- Hugo Chávez regresa a Caracas en la última fase de su enfermedad. (ABC; 19/02/2013)
- Muérase usted, por favor. (ABC; 28/01/2013)
- Hollande abre la puerta a la eutanasia con una futura ley de suicidio asistido. (ABC; 19/12/2012)
- Arranca la huelga de dos días contra la reforma sanitaria. (ABC; 26/11/2012)
- Ramón de Icaza y Zabálburu (1936-2012) Histórico consejero del Bilbao. (ABC; 25/10/2012)
- Botella inaugura un centro de enfermedades avanzadas. (ABC; 12/10/2012)
- Solidaridad familiar ante las graves dolencias. (ABC; 12/09/2012)
- La libertad condicional de enfermos incurables. (ABC; 26/08/2012)
- Conversaciones interceptadas a presos etarras revelan la farsa de su ayuno. (ABC; 16/08/2012)
- La huelga de hambre desvela tensión entre los presos de ETA. (ABC; 13/08/2012)
- Belén Ordóñez El discreto adiós de la hermana de Carmina. (ABC; 04/08/2012)
- Un "carcelero" de Ortega Lara, al hospital del San Sebastián. (ABC; 02/08/2012)
- Ver fútbol reduce el dolor de los pacientes terminales. (ABC; 13/06/2012)
- The Salvation Army visita la Fundación "La Caixa". (ABC; 06/06/2012)
- Las propuestas de los candidatos. (ABC; 06/05/2012)
- En Francia, una primera vuelta muy complicada. (ABC; 24/04/2012)
- Las cinco cosas de las que nos arrepentimos antes de morir. (ABC; 21/12/2011)
- Eutanasia y PSOE. (ABC; 30/08/2011)
- Retiran la alimentación a una enferma sin testamento vital. (ABC; 25/08/2011)
- ¿Qué hará el PP si accede al gobierno? (ABC; 08/08/2011)
- Estado de depresión. (ABC; 28/06/2011)
- "Algunos aspectos de la ley de 'muerte digna' son proeutanásicos". (ABC; 24/06/2011)
- Vida y muerte. (ABC; 13/06/2011)
- Eutanasia encubierta. (ABC; 12/06/2011)
- El gobierno da luz verde a la ley de muerte digna. (ABC; 11/06/2011)
- Jack Kervorkian El final del "doctor muerte". (ABC; 04/06/2011)
- Un proyecto de ingeniería social . (ABC; 26/05/2011)
- Eutanasia. (ABC; 22/05/2011)
- Ya hay muerte digna. (ABC; 16/05/2011)
- ¿Seguridad jurídica o paso a la eutanasia? (ABC; 14/05/2011)
- Muerte digna o eutanasia. (ABC; 14/05/2011)
- La ley de Muerte Digna no permitirá la objeción de conciencia médica. (ABC; 13/05/2011)
- Calidad de vida hasta el final. (ABC; 08/05/2011)
- El congreso languidece ante la escasa iniciativa del Gobierno. (ABC; 24/04/2011)
- Pajín anuncia para mayo la polémica ley de muerte digna. (ABC; 06/04/2011)
- Un año de agonía legislativa. (ABC; 04/04/2011)
- Un clamor por la vida. (ABC; 27/03/2011)
- Por qué acudir al acto "¡Sí a la vida!". (ABC; 25/03/2011)
- Las comunidades socialistas avivan el debate de la muerte digna y la eutanasia. (ABC; 25/03/2011)
- El miedo al dolor, el mayor temor. (ABC; 25/03/2011)
- "Nadie al borde de la muerte me ha pedido la eutanasia". (ABC; 24/02/2011)
- Francia entierra la Ley de Eutanasia / Un debate no cerrado en España. (ABC; 26/01/2011)
- Los médicos piden ayudas exprés para enfermos terminales. (ABC; 19/01/2011)
- "El debate de la eutanasia no se ha agotado con la ley de muerte digna". (ABC; 28/11/2010)
- El PSOE admite que el "caso montes" inspira su ley de la "muerte digna". (ABC; 23/11/2010)
- El gobierno se lanza a regular por ley la "muerte digna". (ABC; 20/11/2010)
- "Me sobrecoge la fortaleza de los que se enfrentan al fin de la vida". (ABC; 20/10/2010)
- Enfermos terminales atendidos 24 horas. (ABC; 17/07/2010)
- "La ELA es una enfermedad incurable y devastadora". (ABC; 31/05/2010)
- La "muerte digna", en vigor en Andalucía a finales de mes. (ABC; 08/05/2010)
- "Ahora sé lo duro que es estar en la Unidad de Dolor de un hospital". (ABC; 19/03/2010)
- El congreso rechaza abrir el debate sobre el "derecho a la eutanasia". (ABC; 10/03/2010)
- Personal del Clínico teme un "caos" en el traslado por obras. (ABC; 12/10/2009)
- Los médicos reclaman más cuidados paliativos para evitar la eutanasia. (ABC; 07/10/2009)
- "La sociedad debe unirse en un frente común contra el cáncer” (ABC; 29/09/2009)
- Máster de Bioética y Bioderecho de la Universidad Rey Juan Carlos. (ABC; 04/09/2009)
- El Parlamento catalán acuerda debatir la eutanasia. (ABC; 19/06/2009)
- Andalucía forzará a los médicos a aplicar su ley de "muerte digna". (ABC; 10/06/2009)
- Inmaculada Echavarría cumplió su deseo de ser desconectada. (ABC;10/06/2009)
- El triplete del no Passssa Nada. (ABC; 31/05/2009)
- "Por la puerta del testamento vital puede colarse la eutanasia". (ABC; 14/03/2009)
- De los cuidados paliativos a la legalización de la eutanasia. (ABC; 09/03/2009)
- "En España aún mueren pacientes con unos sufrimientos evitables". (ABC-Sevilla; 21/01/2014)
- I Curso de formación en Cuidados Paliativos. (ABC-Sevilla; 05/11/2013)
- Tres mil pacientes reciben paliativos cada año. (ABC-Sevilla; 02/11/2013)
- Redes asistenciales. (ABC-Sevilla; 12/10/2013)
- Obstinación terapéutica, un tormento al paciente terminal. (ABC-Sevilla; 22/03/2013)
- El Año Jubilar Macareno tendrá obra social y un congreso mariológico internacional. (ABC-Sevilla; 10/03/2013)
- Investigación y leyes contra el tabaco, armas letales para el cáncer de pulmón. (ABC-Sevilla; 22/11/2012)
- Arenas pide una auditoría de las facturas de la Junta. (ABC-Sevilla; 20/01/2012)
- El documental "Alma", premio del Cicus. (ABC-Sevilla; 12/11/2011)
- Los últimos días de Ramona. (ABC-Sevilla; 12/09/2011)
- Vivir con dignidad y morir en paz. (ABC-Sevilla; 06/09/2011)
- Derecho a Vivir acusa a la Junta de "dejar de alimentar" a una anciana. (ABC-Sevilla; 26/08/2011)
- "La Consejería no es nadie para dar una orden de este tipo". (ABC-Sevilla; 25/08/2011)
- Rubalcaba avisa de que su primera ley será la que regule la "muerte digna". (ABC-Sevilla; 23/08/2011)
- "Al Gobierno andaluz le hace falta la Divina Providencia para pagar sus deudas". (ABC-Sevilla; 07/08/2011)
- Cuidados paliativos, ya. (ABC-Sevilla; 22/06/2011)
- El gobierno da luz verde a la ley de muerte digna. (ABC-Sevilla; 11/06/2011)
- Un proyecto de ingeniería social . (ABC-Sevilla; 26/05/2011)
- Y se prepara el cierre de camas en el Duque del Infantado. (ABC-Sevilla; 12/05/2011)
- Calidad de vida hasta el final. (ABC-Sevilla; 08/05/2011)
- Banderas para todo. (ABC-Sevilla; 02/04/2011)
- Sevilla también se echó a la calle para protestar contra el aborto y cualquier atentado a la vida. (ABC-Sevilla; 28/03/2011)
- Por qué acudir al acto "¡Sí a la vida!". (ABC-Sevilla; 25/03/2011)
- El miedo al dolor, el mayor temor. (ABC-Sevilla; 25/03/2011)
- Pabellón vasco. (ABC-Sevilla; 03/03/2011)
- "Los adolescentes mejoran cuando se tratan en unidades pediátricas". (ABC-Sevilla; 16/02/2011)
- Manipulación semántica y hartazgo ciudadano. (ABC-Sevilla; 01/12/2010)
- "El debate de la eutanasia no se ha agotado con la ley de muerte digna". (ABC-Sevilla; 28/11/2010)
- La Parca, de nuevo en Sevilla. (ABC-Sevilla; 05/11/2010)
- La US acoge la Asamblea Estatal de Estudiantes de Medicina. (ABC-Sevilla; 25/10/2010)
- Expertas piden recursos para luchar contra el cáncer infantil. (ABC-Sevilla; 22/10/2010)
- Atención domiciliaria. (ABC-Sevilla; 12/10/2010)
- Expertos reclaman unidades de Cuidados Paliativos Pediátricos. (ABC-Sevilla; 09/10/2010)
- La "muerte digna", en vigor en Andalucía a finales de mes. (ABC-Sevilla; 08/05/2010)
- Una gran labor asistencial. (ABC-Sevilla; 26/04/2010)
- Protesta laboral por la reconversión del hospital Duque del Infantado. (ABC-Sevilla; 21/04/2010)
- Hermano Francisco Simón - El limosnero de Sevilla. (ABC-Sevilla; 17/04/2010)
- ¡Muerte digna, sí, Eutanasia, no! (ABC-Sevilla; 24/03/2010)
- El Hospital San Lázaro, lleno de gritas y con riesgo de desprendimientos. (ABC-Sevilla; 22/03/2010)
- Aprobada la ley de muerte digna pese a faltar habitaciones individuales para casos terminales. (ABC-Sevilla; 18/03/2010)
- El 20% del área hospitalaria Macarena cuenta con habitaciones individuales. (ABC-Sevilla; 02/02/2010)
- Los médicos reprochan a la Junta que vete la objeción de conciencia a la muerte digna. (ABC-Sevilla; 18/11/2009)
- Joaquín Torres releva a Pilar Serrano en la gerencia del Virgen Macarena. (ABC-Sevilla; 05/11/2009)
- Día mundial de los cuidados paliativos. (ABC-Sevilla; 12/10/2009)
- Aval del Gobierno a la Ley de muerte digna. (ABC-Sevilla; 08/10/2009)
- "Es una barbaridad querer configurar al hombre a base de mayorías". (ABC-Sevilla; 22/06/2009)
- El Parlamento catalán acuerda debatir la eutanasia. (ABC-Sevilla; 19/06/2009)
- Los médicos reclaman poder opinar sobre la Ley de Muerte Digna. (ABC; 17/06/2009)
- La ley de "muerte digna" excluye el derecho a objeción de conciencia médica. (ABC-Sevilla; 10/06/2009)
- El triplete del no Passssa Nada. (ABC-Sevilla; 31/05/2009)
- Sevilla concederá la Medalla de la Ciudad a los cuatro alcaldes de la democracia. (ABC-Sevilla; 11/05/2009)
- "Las mujeres hacemos falta también en la Iglesia; con el tiempo habrá obispas. (ABC-Sevilla; 31/03/2009)
- Premio a la mejor comunicación. (ABC-Sevilla; 25/03/2009)
- El hospital de San Lázaro se adapta a la "inminente" Ley de Muerte Digna. (ABC-Sevilla; 10/03/2009)
- Nueva unidad de cuidados paliativos en San Lázaro. (ABC-Sevilla; 10/03/2009)
- La voz de Sevilla. (ABC-Sevilla; 07/03/2009)
- Recibe un certificado de calidad. (ABC-Sevilla; 11/02/2009)
- La inversión privada en sanidad supera ya la de las obras paralizadas de la Junta. (ABC-Córdoba; 13/11/2013)
- Satse denuncia la eliminación de puestos de trabajo. (ABC-Córdoba; 27/06/2013)
- La obra de la residencia del Sociosanitario empezará en mayo. (ABC-Córdoba; 31/01/2012)
- Cuidados paliativos para 1.400 personas. (ABC-Córdoba; 26/01/2012)
- Satse denuncia la supresión de los cuidados paliativos en la comarca. (ABC-Córdoba; 22/11/2011)
- San Juan de Dios inicia la obra que modernizará sus servicios. (ABC-Córdoba; 26/10/2011)
- San Juan de Dios ofrecerá tratamiento oncológico completo. (ABC-Córdoba; 08/07/2011)
- Un millar de personas se congrega en defensa de la vida. (ABC-Córdoba; 27/03/2011)
- Una plataforma demanda políticas para embarazadas y enfermos. (ABC-Córdoba; 25/03/2011)
- San Juan de Dios se amplía en 80 camas, ocho quirófanos y el triple de consultas. (ABC-Córdoba; 09/03/2010)
- Salud insiste en no regular la objeción de conciencia de los médicos en la Ley de Muerte Digna. (ABC-Córdoba; 09/07/2009)

***El País***

- Un paso más hacia una muerte digna. (El País; 01/06/2013)
- El primer centro privado holandés para eutanasia tiene lista de espera. (El País; 17/03/2013)
- Francia abre la puerta al suicidio asistido de enfermos terminales. (El País; 19/12/2012)
- "Si me quedase embarazada de otro bebé tan enfermo iría a abortar fuera". (El País; 26/07/2012)
- Peter Goodwin, médico que abrió el camino al final digno. (El País; 23/03/2012)
- "No agradezco al médico estar vivo". (El País; 30/01/2012)
- "Lo que Pedro pedía era una eutanasia y eso no es posible". Regular el bien morir, tarea pendiente. (El País; 23/12/2011)
- "Vivo en una cárcel que se estrecha". Un agujero en la ley. (El País; 22/12/2011)
- Una guía médica regula la sedación paliativa. (El País; 11/11/2011)
- Lo social y las distracciones. (El País; 29/10/2011)
- Las cesiones del gobierno. (El País; 14/08/2011)
- Enfermos terminales y cuidados paliativos. (El País; 09/08/2011)
- Madrid elude su deber de garantizar paliativos a la anciana de Leganés. (El País; 03/08/2011)
- No hay muerte digna para Carmen López. (El País; 02/08/2011)
- Denunciada una residencia por no aplicar paliativo a una anciana. (El País; 02/08/2011)
- Cuidado o tratamiento. (El País; 02/08/2011)
- Los médicos de cuidados paliativos apoyan la ley. (El País; 17/05/2011)
- Morir sin dolor. (El País; 16/05/2011)
- ¿Quién teme a la eutanasia? (El País; 15/05/2011)
- Cala la cultura de la humanización del fallecimiento. (El País; 14/05/2011)
- El consejo de ministros aprueba hoy la ley de muerte digna. (El País; 13/05/2011)
- Insultos para la audiencia. (El País; 27/04/2011)
- El paciente sufre las esperas y la descoordinación. (El País; 15/04/2011)
- Más geriatras para descongestionar las urgencias de los hospitales. (El País; 29/03/2011)
- Fumar hasta el final. (El País; 21/03/2011)
- El PSOE intenta cerrar la puerta a la eutanasia en el congreso. (El País; 09/02/2011)
- Francia reabre el debate de la eutanasia más allá de las ideologías. España limita la discusión a los cuidados paliativos. (El País; 26/01/2011)
- Un cáncer del páncreas curado gracias al trasplante a un ratón. (El País; 11/01/2011)
- El PP bautiza 2010 como el "año horrible" de la sanidad. (El País; 21/12/2010)
- La lista de espera quirúrgica baja a 61 días, la menor registrada. (El País; 10/12/2010)
- Jáuregui plantea a Rouco que la Iglesia respete la soberanía popular. (El País; 08/12/2010)
- Quiero morir en casa. ¿Puedo? (El País; 03/12/2010)
- Morir con dignidad. (El País; 20/11/2010)
- Zapatero retoma el impulso social para agotar la legislatura en 2012. (El País; 20/11/2010)
- Una ley para la muerte digna blindará la acción de los médicos. (El País; 20/11/2010)
- Un 29% de los dirigentes médicos se declara a favor de la eutanasia. (El País; 18/11/2010)
- "Haré cumplir la ley del tabaco con los instrumentos del Estado" . (El País; 12/11/2010)
- "¿Verdades? Solo cuando las pida el enfermo". (El País; 21/09/2010)
- España suspende en paliativos. (El País; 16/08/2010)
- Nada más allá de los cuidados paliativos. (El País; 19/06/2010)
- Sanidad centralizará las compras de vacunas para la gripe. (El País; 01/06/2010)
- La subjetividad define qué enfermo es terminal. (El País; 06/04/2010)
- Si hay que morir, mejor saberlo. (El País; 22/03/2010)
- Paliativos contra el dolor sordo. (El País; 02/03/2010)
- Poca atención a los niños en cuidados paliativos. (El País; 10/10/2009)
- Unos 2000 enfermos terminales al año piden la eutanasia. (El País; 07/10/2009)
- La justicia de Australia acepta que se deje de alimentar a un tetrapléjico. (El País; 15/08/2009)
- 56.000 personas registran su voluntad de morir dignamente. (El País; 13/08/2009)
- La eutanasia filmada que nunca existió. (El País; 21/06/2009)
- Aún se muere sufriendo . (El País; 21/06/2009)
- Lamela: "Jamás insinué que hubiera eutanasia en Leganés". (El País; 18/06/2009)
- El derecho a morir en paz. (El País; 17/06/2009)
- Asturias y Cataluña se interesan por la regulación andaluza de la muerte digna. (El País; 16/06/2009)
- El PP sólo apoyará la ley de muerte digna si respeta las "creencias”. Explíquenselo a la ministra. (El País; 11/06/2009)
- Andalucía regula por ley el derecho del enfermo terminal a la sedación. (El País; 10/06/2009)
- Guía audiovisual para atención a enfermos terminales. (El País; 09/06/2009)
- Sólo hay 400 unidades de paliativos para 200.000 enfermos terminales. (El País; 29/05/2009)
- "Los males de la sanidad no son por falta de médicos". (El País; 12/05/2009)
- Muerte digna. (El País; 23/04/2009)
- "El aborto está socialmente superado". (El País; 20/04/2009)
- Veinte meses con muchas propuestas y poco que hacer. (El País; 08/04/2009)
- ¿Dónde está el Dr. House? (El País; 13/03/2009)
- España también deja rendijas para una Eluana. (El País; 13/02/2009)
- Víctima. (El País; 02/02/2009)
- El corto camino al Oscar . (El País; 09/01/2009)

***El Mundo***

- España, huérfana de cuidados paliativos. (El Mundo; 17/02/20014)
- Bélgica legaliza la eutanasia infantil. (El Mundo; 14/02/2014)
- La metástasis del sistema alcanza a los "con papeles". (El Mundo; 17/06/2013)
- La Unidad de Abrazos Paliativos. (El Mundo; 02/04/2013)
- El PSOE agita viejos fantasmas. (El Mundo; 07/02/2012)
- Investigan una millonaria web cedida a un ex alto cargo de Vara. (El Mundo; 06/11/2011)
- Joe Frazier tiene cáncer de hígado. (El Mundo; 06/11/2011)
- La quiniela de… Federico Trillo. (El Mundo; 04/11/2011)
- El rastro del fantasma de Lady Di. (El Mundo; 27/04/2011)
- El sentido de la muerte y de la vida. (El Mundo; 02/02/2011)
- Las unidosis llegarán a España en enero. (El Mundo; 10/12/2010)
- Rubalcaba dice que dentro de cuatro meses se aprobará la Ley sobre la Muerte Digna. (El Mundo; 20/11/2010)
- 37 millones para cuidados paliativos. (El Mundo; 17/07/2010)
- Rivas promueve su Asesoría por una Muerte Digna. (El Mundo; 01/11/2009)
- Reforma legal sobre el suicidio asistido. (El Mundo; 30/10/2009)
- El sacerdote al que pretendió la CIA. (El Mundo; 27/10/2009)
- Dulce, odioso, desafiante, genial. (El Mundo; 30/08/2009)
- La enfermera de Rayan. (El Mundo; 18/07/2009)
- La muerte digna. (El Mundo; 19/06/2009)
- Andalucía aprueba la primera ley española sobre muerte digna. (El Mundo; 10/06/2009)
- Ignacio graba el adiós. (El Mundo; 29/03/2009)
- Morir muerto. (El Mundo; 01/03/2009)
- Especializados en mitigar el dolor de los que más sufren. (El Mundo; 18/01/2009)

*Online edition*

- La justicia se opone a la eutanasia de un enfermo en estado vegetativo. (Elmundo.es; 16/01/2014)
- ¿Alguna vez has pensado que hace Papá Noel en verano? (Elmundo.es; 05/12/2013)
- Los cuidadores tienen mayor esperanza de vida. (Elmundo.es; 21/10/2013)
- Tirar la piedra y esconder la mano. (Elmundo.es; 14/10/2013)
- Derecho a Vivir dice que el PSIB pretende “colar” la eutanasia en Baleares. (Elmundo.es; 27/08/2013)
- El PSIB presenta una iniciativa sobre la “muerte digna”. (Elmundo.es; 26/08/2013)
- Son Espases hace sesiones para dar una respuesta humanizada al cáncer infantil. (Elmundo.es; 06/08/2013)
- El poder de la música en la UCI. (Elmundo.es; 21/05/2013)
- Los médicos franceses tienden la mano a una ley sobre la eutanasia. (Elmundo.es; 14/02/2013)
- El ministro de Finanzas japonés pide a los ancianos que “se den prisa en morir”. (Elmundo.es; 22/01/2013)
- Los cuidados paliativos, “víctimas” de los recortes sanitarios en Madrid. (Elmundo.es; 11/01/2013)
- Francia da el primer paso para legislar sobre la eutanasia. (Elmundo.es; 18/12/2012)
- ¿Cómo se cuida a los cardiópatas al final de la enfermedad? (Elmundo.es; 27/11/2012)
- Una fundación que asiste a enfermos sin cura pide con urgencia voluntarios. (Elmundo.es; 10/09/2012)
- El hospital te enseña a ser paciente. (Elmundo.es; 26/08/2012)
- Los pobres tienen más miedo a la muerte. (Elmundo.es; 16/08/2012)
- El Parlamento Vasco acepta debatir sobre los derechos a una “muerte digna”. (Elmundo.es; 28/06/2012)
- Volver a la vida con la Eurocopa. (Elmundo.es; 18/06/2012)
- Ver fútbol disminuye en un 8% la intensidad del dolor en los pacientes. (Elmundo.es; 13/06/2012)
- Palencia pone en marcha el observatorio de Cuidados Paliativos de Castilla y León. (Elmundo.es; 12/06/2012)
- Por una ley estatal de atención al final de la vida. (Elmundo.es; 29/05/2012)
- Los médicos de cuidados paliativos reivindican su especialidad contra la crisis. (Elmundo.es; 11/05/2012)
- El congreso rechaza despenalizar la asistencia a morir a un enfermo terminal. (Elmundo.es; 27/03/2012)
- ¿De qué se arrepentiría antes de morir? (Elmundo.es; 01/02/2012)
- El SUMMA atenderá a los pacientes de cuidados paliativos por teléfono. (Elmundo.es; 29/11/2011)
- El cáncer sale muy caro. (Elmundo.es; 28/09/2011)
- El Senado argentino comienza a debatir leyes sobre una muerte digna. (Elmundo.es; 28/09/2011)
- La droga que anula la depresión. (Elmundo.es; 28/09/2011)
- Un médico nos obligó a sondar a mi madre en contra de su voluntad. Fue un chantaje. (Elmundo.es; 24/08/2011)
- Los médicos regulan la objeción de conciencia. (Elmundo.es; 12/07/2011)
- El dolor sigue siendo un problema mal manejado. (Elmundo.es; 25/06/2011)
- El consuelo del cine. (Elmundo.es; 18/06/2011)
- Los profesionales piden el máximo consenso político para la ley de muerte digna. (Elmundo.es; 13/05/2011)
- Leire Pajín asegura que la Ley de Muerte Digna no despenalizará la eutanasia. (Elmundo.es; 13/05/2011)
- Los cuidados paliativos se olvidan de los niños. (Elmundo.es; 14/04/2011)
- Navarra aprueba por unanimidad una ley para la muerte digna propuesta por NaBai. (Elmundo.es; 17/03/2011)
- ¿Es ésta la sanidad que nos espera? (Elmundo.es; 02/02/2011)
- Cómo mueren los españoles. (Elmundo.es; 19/12/2010)
- El 93% de la provincia dispondrá de atención sanitaria domiciliaria. (Elmundo.es; 11/12/2010)
- ¿Qué dicen nuestros vecinos europeos de la muerte digna? (Elmundo.es; 19/11/2010)
- El Gobierno anuncia una ley de muerte digna. (Elmundo.es; 19/11/2010)
- Prohibido morirse. (Elmundo.es; 24/03/2010)
- El optimismo mediático del cáncer. (Elmundo.es; 17/03/2010)
- El acceso actual a la morfina en Europa del este es 'una catástrofe'. (Elmundo.es; 25/02/2010)
- Aliviar el espíritu de los enfermos. (Elmundo.es; 28/11/2009)
- Los padres de 'Baby RB' acuerdan que debe morir. (Elmundo.es; 10/11/2009)
- Suiza quiere dejar de ser un destino del 'turismo suicida' en Europa. (Elmundo.es; 28/10/2009)
- Catorce estados pueden utilizar el cannabis con fines terapéuticos en EEUU. (Elmundo.es; 20/10/2009)
- A la cola en recursos de cuidados paliativos y con incompleta cobertura. (Elmundo.es; 09/10/2009)
- Los dispositivos de cuidados paliativos han aumentado en España. (Elmundo.es; 09/10/2009)
- La ley andaluza que regula la muerte digna recibe el primer aval del Parlamento. (Elmundo.es; 07/10/2009)
- Carta póstuma de una doctora con cáncer. (Elmundo.es; 23/07/2009)
- Piden una formación especial a los médicos para aplicar la Ley de Muerte Digna. (Elmundo.es; 23/06/2009)
- El Consejo de Gobierno aprueba el proyecto de ley de Muerte Digna. (Elmundo.es; 09/06/2009)
- Ignacio graba su adiós. (Elmundo.es; 29/03/2009)
- Una nueva unidad de cuidados paliativos atenderá a 1.500 pacientes al año. (Elmundo.es; 09/03/2009)
- Joaquín Torres releva a Pilar Serrano como gerente del hospital Virgen Macarena. (Elmundo.es; 04/11/2009)

***La Vanguardia***

- La Universitat de Vic prevé abrir facultad de Medicina. (La Vanguardia; 02/12/2013)
- ¿Es necesario sufrir para morir? (La Vanguardia; 27/10/2013)
- Stephen Hawking apoya el derecho al suicidio asistido. (La Vanguardia; 18/09/2013)
- Salut quiere dar cuidados paliativos a los enfermos crónicos graves. (La Vanguardia; 23/01/2013)
- Baleares rectifica y decide mantener dos hospitales. (La Vanguardia; 25/11/2012)
- "El dolor no tiene sentido, lo único que sí lo tiene es intentar mitigarlo". (La Vanguardia; 28/06/2012)
- Down+Alzheimer. (La Vanguardia; 06/02/2012)
- "Los pacientes oncológicos atendidos en equipos multidisciplinares tienen mayor supervivencia". (La Vanguardia; 04/02/2012)
- Orfandad 'queer' -Paula Ettelbrick (1955-2011). (La Vanguardia; 05/11/2011)
- Zapatero y Rajoy se miden en un momento dramático para Europa. (La Vanguardia; 28/06/2011)
- Los obispos instan a desobedecer la ley de muerte digna si no se modifica. (La Vanguardia; 28/06/2011)
- Curarse un cáncer a los 80. (La Vanguardia; 14/06/2011)
- El cardenal Rouco afirma que la ley de la muerte digna "no es eutanasia" . (La Vanguardia; 27/05/2011)
- Los enfermos terminales deberán disponer de habitación individual. (La Vanguardia; 14/05/2011)
- El Gobierno busca votos con la ley de muerte digna antes del 22-M. (La Vanguardia; 30/04/2011)
- La vida en campaña. (La Vanguardia; 08/04/2011)
- Los obispos se centran ahora en arremeter contra la eutanasia. (La Vanguardia; 17/03/2011)
- La ley de muerte digna seguirá los pasos de la norma andaluza. (La Vanguardia; 27/12/2010)
- Los cuidados paliativos se extienden también a los niños . (La Vanguardia; 10/12/2010)
- El cardenal Amigo defiende los cuidados paliativos. (La Vanguardia; 23/11/2010)
- La muerte digna, por ley. ¿Qué pesa más, el temor a sufrir o a morir? (La Vanguardia; 20/11/2010)
- ¿Qué pesa más el temor a sufrir o a morir? (La Vanguardia; 20/11/2010)
- La muerte digna, por ley. (La Vanguardia; 20/11/2010)
- El gobierno lanza una ofensiva de leyes hasta junio. (La Vanguardia; 20/11/2010)
- Clínica Coroleu. (La Vanguardia; 11/11/2010)
- Doctor, ¿me muero? (La Vanguardia; 03/10/2010)
- La sociedad por delante. (La Vanguardia; 28/08/2010)
- El cáncer colorrectal se consolida como el más frecuente en Catalunya. (La Vanguardia; 13/07/2010)
- "¡Intentaré que la muerte me encuentre bien vivo!". (La Vanguardia; 10/04/2010)
- Aprende a vivir, lo que dure. (La Vanguardia; 30/11/2009)
- "Hay que entender qué quiere el paciente". (La Vanguardia; 30/11/2009)
- Apoyo integral a personas con enfermedades avanzadas. 08/11/2009)
- Todos contra el Alzheimer. (La Vanguardia; 26/08/2009)
- Muere la anciana que inició una huelga de hambre para que le aplicasen la eutanasia. (La Vanguardia; 02/04/2009)
- Ofensiva para ampliar el apoyo a pacientes terminales. (La Vanguardia; 28/03/2009)
- Sebastià Serrano invita en su nuevo libro a descubrir el papel de los sentidos. (La Vanguardia; 26/03/2009)
- Decisiones personales. (La Vanguardia; 15/02/2009)
- No sólo de curas vive el enfermo terminal. (La Vanguardia; 12/01/2009)

*Online edition*

- La esposa de un enfermo vegetativo recurre la sentencia que impide su muerte. (Lavanguardia.com; 28/01/2014)
- Salud atendió el año pasado a 2.530 pacientes través de sus equipos de Cuidados Paliativos, el 6,5% más que en 2012. (Lavanguardia.com; 20/01/2014)
- Un proyecto pedagógico se propone mostrar a los escolares navarros la muerte como un proceso natural. (Lavanguardia.com; 26/12/2013)
- Más de 200 profesionales debatirán sobre la atención en cuidados paliativos. (Lavanguardia.com; 12/12/2013)
- Enfermeras piden dar cuidados paliativos a enfermos crónicos en ambulatorios. (Lavanguardia.com; 12/12/2013)
- El personal médico y de enfermería de los centros de salud de Mallorca recibirá formación sobre cuidados paliativos. (Lavanguardia.com; 10/12/2013)
- La Plataforma de Atención Específica de Cuidados Paliativos 24 horas atiende casi 15.000 llamadas en lo que va de año. (Lavanguardia.com; 25/11/2013)
- Gustavo Martín Garzo abre mañana las VI Jornadas de Cuidados Paliativos de CyL en el Benito Menni. (Lavanguardia.com; 17/10/2013)
- Más de 100 profesionales del Área Sanitaria Jaén-Norte se forman para mejorar la prestación de cuidados paliativos. (Lavanguardia.com; 17/10/2013)
- Programa de Cuidados Paliativos Pediátrico de la Arrixaca amplía su oferta con la atención a domicilio. (Lavanguardia.com; 11/10/2013)
- Sáez Aguado apuesta por la integración de los profesionales de Atención Primaria en los cuidados paliativos. (Lavanguardia.com; 04/10/2013)
- Los extremeños que reciban cuidados paliativos y necesitan recursos de la Dependencia serán valorados en 15 días. (Lavanguardia.com; 17/07/2013)
- Un total 70 profesionales participan en Menorca en el V Encuentro Balear de Profesionales de Cuidados Paliativos. (Lavanguardia.com; 13/06/2013)
- Salud cede un vehículo para las visitas domiciliarias de cuidados paliativos del Hospital Mateu Orfila de Maó. (Lavanguardia.com; 11/06/2013)
- La Unidad de Cuidados Paliativos del José Molina Orosa recibe el Premio ‘Conejero del año’. (Lavanguardia.com; 08/06/2013)
- Unos 300 voluntarios participan en el Plan de Cuidados Paliativos para acompañar a los pacientes y sus familias. (Lavanguardia.com; 15/04/2013)
- Salud atendió a través de sus equipos de Cuidados Paliativos a cerca de 2.400 pacientes. (Lavanguardia.com; 31/01/2013)
- El Hospital de Son Espases cuenta con una nueva Unidad de Curas Paliativas Pediátricas. (Lavanguardia.com; 30/01/2013)
- Teléfono atención ciudadana de Equipos de Cuidados Paliativos recibe más de 20.000 consultas y atiende a 2.600 pacientes. (Lavanguardia.com; 28/01/2013)
- Más de 500.000 españoles necesitan atenciones paliativas que no reciben. (Lavanguardia.com; 22/01/2013)
- Salud confía en reducir un 15% los ingresos hospitalarios con la atención paliativa a crónicos. (Lavanguardia.com; 22/01/2013)
- Unidad de Cuidados Paliativos del Hospital Doctor José Molina Orosa (Lanzarote) recibe el Premio Ajey. (Lavanguardia.com; 17/12/2012)
- La Comunidad desarrolla un sistema centralizado de información de cuidados paliativos. (Lavanguardia.com; 02/12/2012)
- La AMCP celebra su V Jornada con el lema 'más calidad para el final de la vida'. (Lavanguardia.com; 23/11/2012)
- El XVIII Certamen Internacional ‘Videomed’ exhibirá un documental sobre el Programa de Cuidados Paliativo de Extremadura. (Lavanguardia.com; 10/11/2012)
- El Programa de Cuidados Paliativos de Extremadura atiende a más de 2.100 enfermos en el último año. (Lavanguardia.com; 08/10/2012)
- Expertos en cuidados paliativos dicen que el duelo no es una enfermedad, sino una experiencia humana. (Lavanguardia.com; 29/06/2012)
- Expertos de toda España participarán en Cáceres en las jornadas de Trabajo Social en Cuidados Paliativos. (Lavanguardia.com; 07/06/2012)
- La UVic inaugura una Cátedra de Cuidados Paliativas para difundir la atención integral. (Lavanguardia.com; 29/05/2012)
- La SEMG defiende el papel integrador del médico de familia en el abordaje de los cuidados paliativos. (Lavanguardia.com; 23/05/2012)
- Salud inicia la formación específica de profesionales para el servicio de atención 24 horas de pacientes en paliativos. (Lavanguardia.com; 23/03/2012)
- España necesita el doble de enfermeras especializadas en cuidados paliativos, según un experto. (Lavanguardia.com; 09/02/2012)
- Casi 5.000 personas resolvieron en 2011 sus dudas sobre cuidados paliativos en la web del SMS. (Lavanguardia.com; 07/02/2012)
- El SUMMA 112 presente un manual de actuación para urgencias extrahospitalarias en cuidados paliativos. (Lavanguardia.com; 14/12/2011)
- La Unidad Cuidados Paliativos del Hospital Niño Jesús, Premio ALGOS-Grünenthal, por contribuir al tratamiento del dolor. (Lavanguardia.com; 02/12/2011)
- El SUMMA pondrá en marcha un servicio telefónico para atender a pacientes de cuidados paliativos. (Lavanguardia.com; 28/11/2011)
- Más de 1.200 personas han contactado con el servicio de atención paliativa del hospital San Juan de Dios de Santurtzi. (Lavanguardia.com; 16/11/2011)
- Unas 60.000 personas mueren cada año sin recibir los cuidados paliativos que necesitan. (Lavanguardia.com; 28/10/2011)
- Derecho a Vivir pide este viernes la tutela judicial de los cuidados paliativos de Ramona Estévez. (Lavanguardia.com; 26/08/2011)
- Unas 250.000 personas necesitan al año cuidados paliativos en España. (Lavanguardia.com; 10/03/2011)

**DIGITAL MEDIA**

***Elconfidencial.com***

- La Caixa ampliará a cien hospitales su programa para enfermedades avanzadas. (Elconfidencial.com; 02/07/2014)
- Morir en España: “la mitad de los enfermos terminales no reciben cuidados paliativos”. (Elconfidencial.com; 03/07/2014)
- Lo que se siente al morir, contado por una enfermera de la UCI. (Elconfidencial.com; 28/01/2014)
- Los cinco desafíos que aguardan a Janet Yellen en la presidencia de la Fed. (Elconfidencial.com; 10/10/2013)
- Una fundación de Daniel Day-Lewis reúne tres millones para un hospital. (Elconfidencial.com; 22/08/2013)
- Los santos inocentes de la crisis. (Elconfidencial.com; 22/06/2013)

***Lainformacion.com***

- Sólo una de cada diez personas que necesitan cuidados paliativos los recibe. (Lainformacion.com 28/01/2014)
- El Hospital de Alicante mide el dolor de sus pacientes a través de un registro informatizado. (Lainformacion.com 20/01/2014)
- Salud atendió el año pasado a 2.530 pacientes a través de sus equipos de Cuidados Paliativos, el 6,5% más que en 2012. (Lainformacion.com 20/01/2014)
- El personal médico y de enfermería de los centros de salud de Mallorca recibirá formación sobre cuidados paliativos. (Lainformacion.com 10/12/2013)
- Sanidad reafirma su compromiso de estudiar la puesta en marcha de un Área de Capacitación de Cuidados Paliativos. (Lainformacion.com 27/11/2013)
- Seis sociedades científicas apoyan la puesta en marcha del Área de Capacitación Específica de Cuidados Paliativos. (Lainformacion.com 27/11/2013)
- En España cerca de 130.000 pacientes necesitan cuidados paliativos especiales al final de su vida. (Lainformacion.com 26/11/2013)
- La Plataforma de Atención Específica de Cuidados Paliativos 24 horas atiende 15.000 llamadas en lo que va de año. (Lainformacion.com 25/11/2013)
- Nueve sociedades científicas acuerdan colaborar para mejorar los cuidados paliativos. (Lainformacion.com; 18/11/2013)
- Más de 600 expertos acuden desde este jueves en Toledo a las X Jornadas de la Sociedad Española de Cuidados Paliativos. (Lainformacion.com; 13/11/2013)
- Gustavo Martín Garzo abre este viernes las VI Jornadas de Cuidados Paliativos de CyL en el Benito Menni. (Lainformacion.com; 18/10/2013)
- Más de 100 profesionales del Área Sanitaria Jaén-Norte actualizan sus conocimientos en cuidados paliativos. (Lainformacion.com; 17/10/2013)
- Programa de Cuidados Paliativos Pediátrico de la Arrixaca amplía su oferta con la atención a domicilio. (Lainformacion.com; 11/10/2013)
- SECPAL denuncia que no exista una ley nacional de cuidados paliativos que legisle la atención integral a estos enfermos. (Lainformacion.com; 11/10/2013)
- Uno de cada tres españoles morirá con dolor por falta de cuidados paliativos. (Lainformacion.com; 11/10/2013)
- Junta apuesta por la integración de los profesionales de Atención Primaria en los cuidados paliativos. (Lainformacion.com; 04/10/2013)
- Sáez Aguado apuesta por la integración de los profesionales de Atención Primaria en los cuidados paliativos. (Lainformacion.com; 04/10/2013)
- La AECC otorga al doctor Marcos Gómez Sancho el 'V de Vida 2013' por su trabajo en cuidados paliativos. (Lainformacion.com; 17/09/2013)
- Una fundación de Daniel Day-Lewis reúne tres millones euros para un hospital. (Lainformacion.com; 21/08/2013)
- Los extremeños que reciban cuidados paliativos serán valorados en 15 días. (Lainformacion.com; 17/07/2013)
- OMC y SECPAL manifiestan su "preocupación" sobre el futuro de los cuidados paliativos en España. (Lainformacion.com; 15/07/2013)
- Los médicos piden a Sanidad una formación específica en materia de cuidados paliativos. (Lainformacion.com; 15/07/2013)
- Cuidados paliativos, periodismo 2.0, accidentes laborales o surf, la próxima semana en Cursos de Verano UC. (Lainformacion.com; 23/06/2013)
- Comienza en Menorca el V Encuentro Balear de Cuidados Paliativos. (Lainformacion.com; 14/06/2013)
- La Unidad de Cuidados Paliativos del José Molina Orosa recibe el Premio 'Conejero del año'. (Lainformacion.com; 08/06/2013)
- La Unidad de Cuidados Paliativos del José Molina Orosa recibe el Premio 'Conejero del año'. (Lainformacion.com; 08/06/2013)
- Salud cede un vehículo para las visitas domiciliarias de cuidados paliativos del Hospital Mateu Orfila de Maó. (Lainformacion.com; 11/06/2013)
- El número de mayores y enfermos atendidos por la Fundación FADE se duplica en los primeros cuatro meses del año. (Lainformacion.com; 09/05/2013)
- El Hospital Pare Jofré elabora un protocolo para detectar precozmente casos de riesgo de duelo complicado. (Lainformacion.com; 04/05/2013)
- Un 20% de los pacientes ingresados en grandes hospitales precisan cuidados paliativos. (Lainformacion.com; 29/04/2013)
- Expertos destacan la importancia de los cuidados paliativos para mejorar la vida de personas con patologías incurables. (Lainformacion.com; 24/04/2013)
- Expertos en cuidados paliativos prefieren decidir con el enfermo mientras es posible que atender su voluntad anticipada. (Lainformacion.com; 23/04/2013)
- Unos 300 voluntarios participan en el Plan de Cuidados Paliativos para acompañar a los pacientes y sus familias. (Lainformacion.com; 15/04/2013)
- Especialistas reivindican una acreditación para los profesionales en cuidados paliativos. (Lainformacion.com; 12/04/2013)
- La Sociedad Andaluza de Cuidados Paliativos inaugura en Almería su XI Congreso con especialistas de Madrid y Cataluña. (Lainformacion.com; 10/04/2013)
- La Comunidad potenciará las pruebas respiratorias para aumentar el diagnóstico precoz de la EPOC. (Lainformacion.com; 15/03/2013)
- Experto avisa de que los tratamientos agresivos en fases avanzadas de enfermedad "no aportan mejorías". (Lainformacion.com; 22/02/2013)
- Los médicos franceses proponen una sedación "terminal" en casos excepcionales. (Lainformacion.com; 14/02/2013)
- OMS alerta de que uno de cada dos países no está preparado para prevenir el cáncer. (Lainformacion.com; 04/02/2013)
- Salud atendió a través de sus equipos de Cuidados Paliativos a cerca de 2.400 pacientes. (Lainformacion.com; 31/01/2013)
- Teléfono atención ciudadana de Equipos de Cuidados Paliativos recibe más de 20.000 consultas y atiende a 2.600 pacientes. (Lainformacion.com; 28/01/2013)
- Acusan a la Comunidad de Madrid de recortes y despidos en los servicios de Cuidados Paliativos. (Lainformacion.com; 09/01/2013)
- Un plan nacional de cuidados paliativos ahorraría al estado 500 millones de euros anuales. (Lainformacion.com; 18/12/2012)
- Unidad de Cuidados Paliativos del Hospital Doctor José Molina Orosa (Lanzarote) recibe el Premio AJY. (Lainformacion.com; 17/12/2012)
- Advierten de que no cumplir el plan de crecimiento de unidades de cuidados paliativos "cuesta dinero al SNS". (Lainformacion.com; 17/12/2012)
- Un plan nacional de cuidados paliativos ahorraría al estado 500 millones de euros anuales. (Lainformacion.com; 16/12/2012)
- La Comunidad desarrolla un sistema centralizado de información de cuidados paliativos. (Lainformacion.com; 03/12/2012)
- La SEC denuncia el déficit de formación cardiológica de los profesionales de cuidados paliativos. (Lainformacion.com; 26/11/2012)
- La AMCP celebra su V Jornada con el lema 'más calidad para el final de la vida'. (Lainformacion.com; 23/11/2012)
- El XVIII Certamen Internacional 'Videomed' exhibirá un documental sobre el Programa de Cuidados Paliativo de Extremadura. (Lainformacion.com; 10/11/2012)
- Expertos advierten de que España necesita unos 1.500 médicos especializados en cuidados paliativos. (Lainformacion.com; 24/10/2012)
- Los cuidados paliativos en oncología ahorrarían 500 millones a la Sanidad. (Lainformacion.com; 12/10/2012)
- La Sociedad Española de Cuidados Paliativos pide que esta especialidad sea reconocida. (Lainformacion.com; 11/10/2012)
- Más de 270 pacientes con necesidad de cuidados paliativos reciben atención a través de Salud Responde. (Lainformacion.com; 08/10/2012)
- El Programa de Cuidados Paliativos de Extremadura atiende a más de 2.100 enfermos en el último año. (Lainformacion.com; 08/10/2012)
- El cáncer y las enfermedades crónicas provocan casi la mitad de las muertes en Europa. (Lainformacion.com; 18/09/2012)
- La Obra Social 'la Caixa' presta apoyo en Extremadura a 615 enfermos avanzados. (Lainformacion.com; 20/08/2012)
- Expertos en cuidados paliativos dicen que el duelo no es una enfermedad, sino una experiencia humana. (Lainformacion.com; 29/06/2012)
- Denuncian el cierre de camas de cuidados paliativos en el Campo de Gibraltar y que se incumple la ley andaluza. (Lainformacion.com; 29/06/2012)
- La mitad de los enfermos terminales mejora el ánimo con atención psicosocial. (Lainformacion.com; 27/06/2012)
- El oncólogo Jaime Sanz asegura que la clave para afrontar la muerte es tenerla presente durante la vida. (Lainformacion.com; 25/06/2012)
- Cáceres acoge hoy las I jornadas de trabajo social en cuidados paliativos. (Lainformacion.com; 20/06/2012)
- Expertos de toda España participan en Cáceres en las jornadas de trabajo social en cuidados paliativos. (Lainformacion.com; 19/06/2012)
- Ver un partido de fútbol reduce más del 8 por ciento el dolor de los pacientes oncológicos en fase avanzada. (Lainformacion.com; 12/06/2012)
- Expertos de toda España participarán en Cáceres en las jornadas de trabajo social en cuidados paliativos. (Lainformacion.com; 09/06/2012)
- El jefe de Oncología de Valdecilla, premio a la mejor trayectoria científica y clínica de la Fundación ECO. (Lainformacion.com; 05/06/2012)
- Constituida la comisión gallega para los cuidados paliativos. (Lainformacion.com; 04/06/2012)
- La UVic inaugura una Cátedra de Cuidados Paliativas para difundir la atención integral. (Lainformacion.com; 29/05/2012)
- Expertos piden una ley estatal de atención al final de la vida y que se forme a los profesionales en cuidados paliativos. (Lainformacion.com; 29/05/2012)
- La Sociedad Española de Cuidados Paliativos transmite su apoyo a los profesionales del Hospital General y Joan March. (Lainformacion.com; 24/05/2012)
- La SEMG defiende el papel integrador del médico de familia en el abordaje de los cuidados paliativos. (Lainformacion.com; 23/05/2012)
- El PSOE propone medidas parlamentarias para impulsar los cuidados paliativos en Extremadura. (Lainformacion.com; 14/05/2012)
- Álvaro Gándara, nuevo presidente de la Sociedad Española de Cuidados Paliativos. (Lainformacion.com; 11/05/2012)
- Sólo 3 de cada 10 españoles con enfermedades terminales acceden a cuidados paliativos específicos. (Lainformacion.com; 09/05/2012)
- Menos del 30% de los pacientes terminales acceden a cuidados paliativos específicos. (Lainformacion.com; 09/05/2012)
- Cerca de 1.200 profesionales analizan la atención al final de la vida en un congreso en Badajoz. (Lainformacion.com; 07/05/2012)
- Uruguay revisará atención paliativa con ayuda española tras caso enfermeros. (Lainformacion.com; 11/04/2012)
- Los Equipos regionales de Cuidados Paliativos atendieron a 2.318 nuevos pacientes en 2011. (Lainformacion.com; 26/03/2012)
- Expertos abordan este miércoles en Bilbao los límites legales de los cuidados paliativos y la muerte digna. (Lainformacion.com; 15/02/2012)
- España necesita doblar su número de enfermeras expertas en cuidados paliativos para atender a 115.000 pacientes al año. (Lainformacion.com; 13/02/2012)
- La web del SMS 'www.cuidarypaliar.es', elegida Mejor web institucional de la Región. (Lainformacion.com; 10/02/2012)
- Casi 5.000 personas resolvieron en 2011 sus dudas sobre cuidados paliativos en la web del SMS. (Lainformacion.com; 07/02/2012)
- Expertos nacionales en cuidados paliativos analizan desde este miércoles en Ourense la situación actual de la asistencia. (Lainformacion.com; 25/01/2012)
- SECPAL no considera la posibilidad de que los cuidados paliativos "no salgan adelante" con el nuevo Gobierno. (Lainformacion.com; 22/12/2011)
- El SUMMA elabora un manual de actuación para urgencias extrahospitalarias en cuidados paliativos. (Lainformacion.com; 18/12/2011)
- Sanidad impulsará una atención paliativa basada en una asistencia coordinada entre Primaria y Especializada. (Lainformacion.com; 16/12/2011)
- Unas 60.000 personas mueren sin recibir los cuidados paliativos que necesitan. (Lainformacion.com; 08/12/2011)
- La Unidad Cuidados Paliativos del Hospital Niño Jesús, Premio ALGOS-Grünenthal, por contribuir al tratamiento del dolor. (Lainformacion.com; 02/12/2011)
- El SUMMA pondrá en marcha un servicio telefónico para atender a pacientes de cuidados paliativos. (Lainformacion.com; 29/11/2011)
- Mantener la ilusión ayuda a los enfermos paliativos a evitar la soledad del final de la vida. (Lainformacion.com; 22/11/2011)
- Salud asegura que la asistencia a sus usuarios es de equidad "con independencia del lugar". (Lainformacion.com; 15/11/2011)
- Los expertos elaboran un protocolo de actuación ante el dolor oncológico. (Lainformacion.com; 11/11/2011)
- La OMC lanza una guía de cuidados paliativos para "quitar el miedo" legal y práctico que genera esta atención. (Lainformacion.com; 10/11/2011)
- Premian un trabajo de la EASP sobre trato humano y atención a pacientes infantiles con cáncer terminal. (Lainformacion.com; 02/11/2011)
- El PSCL acusa a Sáez Aguado de incumplir el compromiso de Guisasola sobre el Plan de Cuidados Paliativos. (Lainformacion.com; 26/10/2011)
- La ANIS atribuye la polémica "de algunos sectores" por la ley estatal de Muerte Digna a su "desconocimiento". (Lainformacion.com; 21/10/2011)
- Sanidad impulsa la formación en cuidados paliativos entre los profesionales de Atención Primaria y Hospitalaria. (Lainformacion.com; 08/10/2011)
- Uno de cada sesenta hogares españoles sufre un caso de enfermedad avanzada o terminal al año. (Lainformacion.com; 07/10/2011)
- Experto asegura que los cuidados paliativos son la forma "más humana y barata" de tratar al paciente terminal. (Lainformacion.com; 07/10/2011)
- Medios dan poca información sobre cuidados paliativos del cáncer. (Lainformacion.com; 06/10/2011)
- El Área de Salud de Ibiza y Formentera participa en la IV Jornada Balear de Cuidados Paliativos. (Lainformacion.com; 28/09/2011)
- El mayor congreso europeo sobre el cáncer tendrá lugar en 2014 en Madrid. (Lainformacion.com; 21/09/2011)
- La Agencia Laín Entralgo organiza nuevos cursos para profesionales. (Lainformacion.com; 14/09/2011)
- Los políticos invitados a visitar las UCIs antes de aprobar la Ley de Muerte Digna. (Lainformacion.com; 09/09/2011)
- Derecho a Vivir pide este viernes la tutela judicial de los cuidados paliativos de Ramona Estévez. (Lainformacion.com; 26/08/2011)
- Pajín cree que la ley de cuidados paliativos es "absolutamente necesaria" para evitar sufrimiento innecesario. (Lainformacion.com; 24/08/2011)
- España necesita 3.000 enfermeros en cuidados paliativos. (Lainformacion.com; 09/06/2011)
- HRW denuncia el fracaso de muchos Gobiernos en los cuidados paliativos del cáncer y el sida. (Lainformacion.com; 02/06/2011)
- Los enfermeros quieren más formación en cuidados paliativos. (Lainformacion.com; 19/05/2011)
- Aresa introduce los cuidados paliativos integrales en su cartera de servicios. (Lainformacion.com; 18/05/2011)
- Acuerdo en el fondo pero no en la forma sobre la ley de cuidados paliativos. (Lainformacion.com; 13/05/2011)
- La sociedad de cuidados paliativos quiere una asignatura obligatoria sobre este tratamiento. (Lainformacion.com; 13/05/2011)
- La coordinadora de Política Social del PP dice que lo que los españoles necesitan es una vida digna. (Lainformacion.com; 13/05/2011)
- El Gobierno aprobará hoy la Ley de Muerte Digna. (Lainformacion.com; 13/05/2011)
- El PP pide un plan nacional de cuidados paliativos. (Lainformacion.com; 07/05/2011)
- España tendrá una ley de cuidados paliativos que ahora sólo tienen dos países. (Lainformacion.com; 20/03/2011)
- Leire Pajín afirma que la ley de muerte digna no regula la eutanasia, mitiga el dolor. (Lainformacion.com; 17/03/2011)
- Pajín asegura que la futura ley de muerte digna no regulará la eutanasia. (Lainformacion.com; 26/01/2011)
- Los colegios médicos piden a Pajín "cambios" en cuidados paliativos. (Lainformacion.com; 12/01/2011)
- Primera reunión de expertos para elaborar la ley de cuidados paliativos. (Lainformacion.com; 21/12/2010)
- La Sociedad Europea de Cuidados Paliativos aboga más que por leyes, por medidas concretas con presupuesto. (Lainformacion.com; 27/11/2010)
- La ley de muerte digna, celebrada como un derecho por unos y rechazada como eutanasia por otros. (Lainformacion.com; 19/11/2010)
- Un centenar de profesionales participa en las IV Jornadas de Cuidados Paliativos dedicadas a la atención al duelo (Lainformacion.com; 29/11/2010)
- Sanidade crea una comisión para asesorar al Sergas en materia de cuidados paliativos. (Lainformacion.com; 21/10/2010)
- El Hospital del Sureste forma a profesionales sanitarios en la atención de cuidados paliativos en Oncología. (Lainformacion.com; 19/10/2010)
- Hospital San Juan de Dios de Santurtzi ofrece ayuda a cuidadores y familiares de personas al final de la vida. (Lainformacion.com; 14/10/2010)
- Sanidad presenta la web 'cuidarypaliar.es', destinada a profesionales, familias y pacientes. (Lainformacion.com; 08/10/2010)
- La sociedad de cuidados paliativos denuncia que 125.000 enfermos terminales no recibe la asistencia necesaria. (Lainformacion.com; 08/10/2010)
- El hospital Centro de Cuidados Laguna destaca que hay "mucho desconocimiento" sobre los cuidados paliativos. (Lainformacion.com; 08/10/2010)
- Hoy se celebra el día mundial de los cuidados paliativos. (Lainformacion.com; 08/10/2010)
- La OMC recuerda que los cuidados paliativos son un "derecho y no un privilegio". (Lainformacion.com; 07/10/2010)
- Unas 200.000 personas necesitan recibir cuidados paliativos en España, pero sólo la mitad los recibe, según la SECPAL. (Lainformacion.com; 07/10/2010)
- El equipo psicosocial del Virgen de la Salud de Toledo ha atendido a más de 1.300 personas. (Lainformacion.com; 04/10/2010)
- El Plan de Salud y Bienestar Social 2011-2020 incluirá una "hoja específica" sobre cuidados paliativos. (Lainformacion.com; 23/09/2010)
- Más del 60% de europeos necesitará cuidados paliativos al final de su vida, según expertos. (Lainformacion.com; 20/09/2010)
- El curso de musicoterapia: "cuidados paliativos y oncología" reúne en Teruel a 31 estudiantes y profesionales sanitarios. (Lainformacion.com; 13/09/2010)
- Responsable de cuidados paliativos pide "un mayor esfuerzo" para proporcionar esta atención con "equidad" en España. (Lainformacion.com; 16/08/2010)
- La Junta crea una red de voluntarios para atender a personas en la etapa final de la vida. (Lainformacion.com; 11/08/2010)
- Comunidad contará con un servicio telefónico 24 horas-365 días al año para pacientes que necesiten cuidados paliativos. (Lainformacion.com; 16/07/2010)
- Buena parte del mundo carece de cuidados paliativos. (Lainformacion.com; 14/07/2010)
- El 80% de los terminales reciben cuidados paliativos en Catalunya. (Lainformacion.com; 12/07/2010)
- El Principado cuanta ya con diez equipos de cuidados paliativos en funcionamiento a los que se sumarán dos más. (Lainformacion.com; 10/07/2010)
- Cantabria recibe 153.600 € para cuidados paliativos, seguridad de pacientes, prevención de violencia de género y partos. (Lainformacion.com; 09/07/2010)
- Cada año fallecen en España cerca de 100.000 pacientes por cáncer. (Lainformacion.com; 02/07/2010)
- Expertos piden más cuidados paliativos para pacientes no oncológicos. (Lainformacion.com; 01/07/2010)
- Euskadi recibirá 151.125 euros del Gobierno central para estrategias de cuidados paliativos y violencia de género. (Lainformacion.com; 25/06/2010)
- Cada año unos 1.500 niños padecen enfermedad terminal en España. (Lainformacion.com; 10/05/2010)
- La SECPAL propondrá a las universidades implantar asignaturas sobre cuidados paliativos en sus planes de estudios. (Lainformacion.com; 10/05/2010)
- Los enfermos terminales conscientes de su estado tienen menor sufrimiento. (Lainformacion.com; 07/05/2010)
- Expertos en cuidados paliativos denuncian falta de profesionales para atender a los más de 200.000 enfermos terminales. (Lainformacion.com; 05/05/2010)
- Expertos en cuidados paliativos denuncian falta de profesionales para atender a los más de 200.000 enfermos terminales. (Lainformacion.com; 05/05/2010)
- La sociedad cántabra de cuidados paliativos se presenta hoy en el colegio de médicos. (Lainformacion.com; 21/04/2009)
- CCOO advierte del cierre de 20 camas de paliativos en el Hospital Duque del Infantado. (Lainformacion.com; 20/04/2010)
- Una enfermera de la UN crea la versión en castellano de un test de evaluación para pacientes con cáncer terminal. (Lainformacion.com; 12/04/2010)
- El Hospital Doctor Moliner de Serra (Valencia) realizará sesiones semanales de musicoterapia. (Lainformacion.com; 04/04/2010)
- La Comunidad refuerza con 36 millones la atención a pacientes que necesitan cuidados paliativos. (Lainformacion.com; 18/03/2010)
- Salud atendió en 2009 a casi 2.000 pacientes terminales a través de sus equipos de Cuidados Paliativos. (Lainformacion.com; 16/03/2010)
- Cuidados Paliativos del Hospital Virgen de la Luz (Cuenca) ha atendido a 164 enfermos en sus domicilios. (Lainformacion.com; 15/03/2010)
- La Unidad de Cuidados Paliativos del Hospital de La Candelaria (Tenerife) ha atendido a más de 8.000 pacientes. (Lainformacion.com; 13/03/2010)
- La sedación paliativa es igual de segura y eficaz a domicilio. (Lainformacion.com; 22/02/2010)
- PP defenderá una medida para que los municipios adscritos al Hospital de Montilla tengan cuidados paliativos. (Lainformacion.com; 15/01/2010)
- La OMC pide al Congreso regular los cuidados paliativos y no la eutanasia. (Lainformacion.com; 18/12/2009)
- Sólo el 25% de los 150.000 enfermos terminales reciben cuidados paliativos. (Lainformacion.com; 17/12/2009)
- La AECC formará en Málaga capital a voluntarios para el acompañamiento a enfermos con cuidados paliativos. (Lainformacion.com; 12/12/2009)
- Facultad de Medicina de la UN, primera en impartir la asignatura de Cuidados Paliativos como materia obligatoria. (Lainformacion.com; 10/12/2009)
- La Escuela de Pacientes inaugura en el Hospital Virgen del Rocío (Sevilla) un aula de Cuidados Paliativos. (Lainformacion.com; 10/12/2009)
- Un profesor de la Universidad de Navarra elabora el primer manual en castellano sobre cuidados paliativos. (Lainformacion.com; 09/12/2009)
- Pastor (PP) asegura que España está "razonablemente bien" en paliativos, pero necesita avanzar en investigación. (Lainformacion.com; 01/12/2009)
- PSN propone elaborar un nuevo plan estratégico de cuidados paliativos en la comunidad. (Lainformacion.com; 25/11/2009)
- La muerte digna, los cuidados paliativos o la historia digital protagonizan la XII Jornadas de Admisión Médica. (Lainformacion.com; 15/11/2009)
- Aumenta el rechazo al cuidado de enfermos terminales por familiares. (Lainformacion.com; 27/10/2009)
- Jiménez recuerda que el uso terapéutico del cannabis está autorizado en España en el marco de los cuidados paliativos. (Lainformacion.com; 20/10/2009)
- El Arnau de Vilanova obtiene el reconocimiento de Centro de Excelencia en cuidados paliativos en tratamiento de cáncer. (Lainformacion.com; 18/10/2009)
- Salud elabora una guía para compilar los recursos sanitarios disponibles para los cuidados paliativos. (Lainformacion.com; 13/10/2009)
- La Rioja "aprueba" en la prestación de cuidados paliativos, aunque carece de plan específico, según SECPAL. (Lainformacion.com; 11/10/2009)
- El Hospital Dr. José Molina Orosa (Lanzarote) incrementa un 30,18% las asistencias domiciliarias de cuidados paliativos. (Lainformacion.com; 10/10/2009)
- España duplica sus dispositivos de cuidados paliativos en los últimos 5 años, según la Sociedad de Cuidados Paliativos. (Lainformacion.com; 09/10/2009)
- Los equipos de cuidados paliativos atendieron en 2008 a más de 1.600 pacientes terminales. (Lainformacion.com; 07/10/2009)
- La Organización Médica Colegial apuesta por aplazar el debate sobre la eutanasia. (Lainformacion.com; 06/10/2009)
- Prevenir el cáncer con educación y programas de 'screening' para el diagnóstico precoz, actuales retos de la Oncología. (Lainformacion.com; 02/10/2009)
- Más de 2.000 pacientes fueron atendidos en las cinco unidades hospitalarias de Cuidados Paliativos en 2008. (Lainformacion.com; 24/08/2009)
- Badajoz será la sede en 2012 del IX Congreso Nacional de Cuidados Paliativos, según se decidió en una Junta en Logroño. (Lainformacion.com; 23/08/2009)
- Los médicos aseguran que un desarrollo urgente de los cuidados paliativos minimizaría la demanda de eutanasia en España. (Lainformacion.com; 02/07/2009)
- Salud invertirá 95.000 euros en la reforma de la Unidad de Cuidados Paliativos del Campo de Gibraltar. (Lainformacion.com; 30/06/2009)
- La UHD del Hospital de Elda ofrece cuidados paliativos a enfermos en el hospital y en su domicilio. (Lainformacion.com; 25/06/2009)
- La Sociedad Española de Cuidados Paliativos nombra a Jesús Viguria socio de honor. (Lainformacion.com; 22/06/2009)
- Sanz aboga por el trabajo a domicilio en los cuidados paliativos y destaca el papel de las familias. (Lainformacion.com; 19/06/2009)
- Gobierno de Canarias ofrece cuidados paliativos a 230 pacientes y realiza 3.828 asistencias domiciliarias en Lanzarote. (Lainformacion.com; 18/06/2009)
- La Sociedad de Cuidados Paliativos pide que los derechos de la Ley de Muerte Digna "no se queden en meras intenciones". (Lainformacion.com; 12/06/2009)
- Piden las suficientes inversiones para cumplir la ley de la muerte digna. (Lainformacion.com; 09/06/2009)
- La Junta aprueba la Ley de dignidad ante el proceso de muerte. (Lainformacion.com; 09/06/2009)
- Más de la mitad de los mayores de 65 años necesitarán cuidados paliativos al final de sus días. (Lainformacion.com; 04/06/2009)
- Una guía audiovisual ayudará a familiares de pacientes terminales. (Lainformacion.com; 28/05/2009)
- Más de 330 expertos se reunirán mañana en Gran Canaria en el Congreso Internacional de Cuidados paliativos. (Lainformacion.com; 19/05/2009)
- Piden que los cuidados paliativos sean considerados derechos fundamentales. (Lainformacion.com; 10/05/2009)
- Expertos sanitarios analizan en el Hospital General los cuidados paliativos. (Lainformacion.com; 28/04/2009)

***Libertaddigital.com***

- La guerra contra las drogas condena al dolor a cientos de miles de enfermos de cáncer. (Libertaddigital.com; 28/11/2013)
- Sarkozy, imagen de una campaña a favor de la eutanasia. (Libertaddigital.com; 07/03/2012)
- Lo correcto y lo elegante en el idioma. (Libertaddigital.com; 07/10/2011)
- Pajín exhibe la ley de "muerte digna", el testamento de Zapatero. (Libertaddigital.com; 13/05/2011)
- El PP: "Los españoles necesitan una vida digna hasta el final de sus días". (Libertaddigital.com; 13/05/2011)
- Mar adentro, otra vez. (Libertaddigital.com; 22/11/2010)
- El PP, a Rubalcaba: "Además de ayudar a morir, ayude a vivir dignamente". (Libertaddigital.com; 20/11/2010)
- Y ahora, el "derecho" a la eutanasia. (Libertaddigital.com; 20/11/2010)
- El Parlamento andaluz aprueba la Ley de la Muerte Digna sin medios para morir en una habitación individual. (Libertaddigital.com; 18/03/2010)
- El Gobierno ultima una ley sobre la muerte digna, pero "no es de eutanasia". (Libertaddigital.com; 19/11/2010)
- Denuncian a 7 médicos por la muerte de una mujer que pidió ayuda cinco veces. (Libertaddigital.com; 04/10/2009)
- Los médicos piden desarrollar los cuidados paliativos para minimizar la eutanasia. (Libertaddigital.com; 02/07/2009)
- El País, obligado a rectificar sobre una eutanasia que no existió. (Libertaddigital.com; 23/06/2009)
- La Junta pone de ejemplo las sedaciones de Leganés en la Ley de Muerte Digna. (Libertaddigital.com; 10/06/2009)
- Andalucía obligará a los hospitales religiosos a aplicar la Ley de Muerte Digna. (Libertaddigital.com; 09/06/2009)
- Montes, héroe médico del socialismo. (Libertaddigital.com; 20/04/2009)
- Un entusiasmo muy pasajero. (Libertaddigital.com; 13/03/2009)
- Derecho a morir, licencia para matar. (Libertaddigital.com; 09/07/2008)

***Publico.es***

- Por el derecho a una muerte digna. (Publico.es; 08/03/2014)
- Merkel quiere penalizar el suicidio asistido. (Publico.es; 11/01/2014)
- El senado belga aprueba la eutanasia para menores. (Publico.es; 13/12/2013)
- Uno de cada tres españoles morirá con "dolor intenso" por falta de cuidados paliativos. (Publico.es; 11/10/2013)
- Uruguay revisará atención paliativa con ayuda española tras caso enfermeros. (Publico.es; 11/04/2012)
- Más niños de Inglaterra viven con enfermedades graves: estudio. (Publico.es; 23/03/2012)
- Cuando faltan camas en terapia intensiva, cambian los objetivos. (Publico.es; 16/03/2012)
- La sedación ya es un derecho para pacientes terminales. (Publico.es; 11/11/2011)
- Medios dan poca información sobre cuidados paliativos del cáncer. (Publico.es; 06/10/2011)
- El ala dura de la Iglesia fuerza la crítica a la Muerte Digna. (Publico.es; 23/06/2011)
- El Gobierno mejorará el acceso a sedantes paliativos. (Publico.es; 10/06/2011)
- Atención en centros de cuidados paliativos no adelanta la muerte. (Publico.es; 19/05/2011)
- La muerte digna será un derecho para todos. (Publico.es; 14/05/2011)
- El médico tendrá que respetar la voluntad del paciente terminal. (Publico.es; 13/05/2011)
- La Ley de Muerte Digna no despenalizará la eutanasia. (Publico.es; 13/05/2011)
- La ley de Paliativos estará lista a principios de Mayo. (Publico.es; 06/04/2011)
- Andalucía resuelve casos extremos sin objeciones. (Publico.es; 04/04/2011)
- La atención en casa al paciente terminal se garantizará por ley. (Publico.es; 04/04/2011)
- Aragón aprueba su Ley de Muerte Digna con la oposición del PP. (Publico.es; 25/03/2011)
- Sanidad consensua con las autonomías la muerte digna. (Publico.es; 27/01/2011)
- Decidir la propia muerte. (Publico.es; 20/12/2010)
- "La ley no regulará la eutanasia". (Publico.es; 20/12/2010)
- "La mayor prueba de amor es que dejara de sufrir". (Publico.es; 20/12/2010)
- Calidad de vida hasta la muerte. (Publico.es; 08/12/2010)
- "Sabía que Amelia se moría pero estaba feliz, que es lo importante". (Publico.es; 07/12/2010)
- "En paliativos hay muchas desigualdades". (Publico.es; 22/11/2010)
- El 58% de los españoles cree que se debería regular la eutanasia. (Publico.es; 20/11/2010)
- Una ley regulará la muerte digna, pero no la eutanasia. (Publico.es; 20/11/2010)
- El Gobierno aprobará una ley de muerte digna en marzo de 2011. (Publico.es; 19/11/2010)
- La demencia avanzada es una enfermedad terminal. (Publico.es; 19/10/2009)
- Buena parte del mundo carece de cuidados paliativos: reporte. (Publico.es; 14/07/2010)
- Británicos envejecidos esperan reforma suicidio asistido. (Publico.es; 28/09/2009)
- Luis Montes augura que Europa abrirá el debate sobre la eutanasia en 10 años. (Publico.es; 11/08/2009)
- Ley de vida. (Publico.es; 21/06/2009)
- La Iglesia predica la muerte sin dolor. (Publico.es; 12/06/2009)
- Que me lleven al sur para morir. (Publico.es; 11/06/2009)
- Enfermos terminales que se refugian en fe buscan prolongar vida. (Publico.es; 18/03/2009)
- "Morirás con dolor", el undécimo mandamiento. (Publico.es; 18/03/2009)
- Sofía Loren apadrina una donación de cuadros para centros de cuidados paliativos. (Publico.es; 03/03/2009)
- Losantos, citado como imputado por injuriar al doctor Montes. (Publico.es; 18/02/2009)
- Solo 60.000 españoles registran el modo en que desean morir. (Publico.es; 12/02/2009)
